# Editorial: Optimizing school readiness for children with developmental disabilities

**DOI:** 10.3389/fped.2023.1196934

**Published:** 2023-04-13

**Authors:** Bolajoko O. Olusanya, M. K. C. Nair, Paul Lynch, Mijna Hadders-Algra

**Affiliations:** ^1^ Centre for Healthy Start Initiative, Lagos, Nigeria; ^2^ NIMS-Spectrum-Child Development Research Centre, Thiruvananthapuram, India; ^3^ School of Education, University of Glasgow, Glasgow, United Kingdom; ^4^ University of Groningen, University Medical Center Groningen, Department of Pediatrics, Division of Developmental Neurology and University of Groningen, Faculty of Theology and Religious Studies, Groningen, Netherlands

**Keywords:** school readiness, inclusive education, early childhood development (ECD), early childhood education, developmental disabilities, nurturing care framework (NCF), SDG #4, early detection and intervention

**Editorial on the Research Topic** Optimizing school readiness for children with developmental disabilities

*“The only thing worse than being blind is having sight with no vision”*

∼Helen Keller

**Figure 1:**
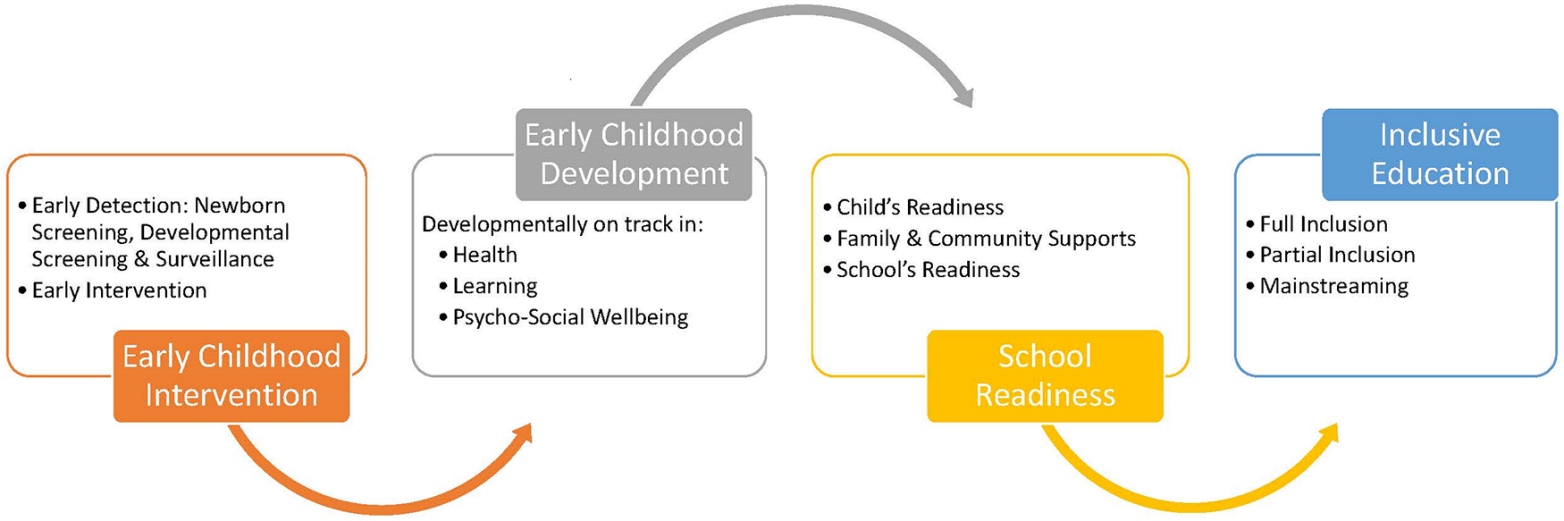
Pathway to early childhood development for inclusive education under the UN Sustainable Development Goals [source: reference (3)].

In September 2015, the 193 Member States of the United Nations (UN) undertook a social contract to advance population health, well-being, and security over the life-course globally under the Sustainable Development Goals (SDGs) (1). This global agenda, for the first time, set out a global vision for early childhood development for children under 5 years with the primary objective of facilitating access to inclusive and equitable quality early childhood education for children with or at risk of developmental disabilities (Figure 1). At that time, when the SDGs were launched, limited data was available on the state of the world's children with developmental delays and disabilities. This data gap was swiftly utilized by WHO, UNICEF and the World Bank to promote an early childhood development program in 2018 tagged the Nurturing Care Framework (NCF), based on an estimated 250 million children suspected to be at risk of poor cognitive development due to stunting and extreme poverty in LMICs (2). However, the NCF was neither geared towards promoting school readiness for inclusive education for children with developmental disabilities as envisioned by the SDGs, nor was it endorsed or accredited as a global program under the SDGs (3).

Emerging data since 2018 has now shown that every day, some 145,000 babies are born with or acquire lifelong disability in early childhood (4). The likelihood of a child being disabled is estimated to be ten-fold than of dying before the fifth birthday (5). A landmark report from UNICEF in 2021 further showed that, compared to children without disabilities, children with disabilities are significantly less likely to have foundational reading and numeracy skills, more likely to have never attended school and are more likely to be out of primary school (6). Lack of formal and quality education places children with developmental disabilities at greater risk of not securing gainful employment and at a higher risk of social exclusion and isolation. This trajectory challenges the moral justice of an exclusive focus on child survival since the era of the Millennium Development Goals (2000–2015) at the expense of a just and equitable society where no one is left behind.

The SDGs global vision to prepare every child, particularly those with developmental disabilities, to receive the best possible education to succeed in life beyond survival inspired the launch of this research topic by the Global Research on Developmental Disabilities Collaborators (GRDDC). GRDDC is a diversified, cross-cultural, and inclusive consortium of professional care providers and parents with and without lived experience of disability dedicated to advancing optimal development for children under 5 years with disabilities. A total of ten papers by 64 authors from all submissions were published drawn from sub-Saharan Africa, South Asia, Latin America, North America, and Europe.

Three papers in this series by Olusanya et al. set out to summarize the available data on children and adolescents with disabilities. The first GRDDC paper (Olusanya et al.) analyzed the latest prevalence estimates of children and adolescents with disabilities reported by UNICEF and the Global Burden of Disease (GBD), two leading publishers of population health metrics for policy makers in global health. The most striking and overarching finding was that the available prevalence estimates of disabilities among children and adolescents generated using either functional approach or statistical modelling can be statistically regarded as comparable and complementary. The choice between these sources is therefore, likely to be guided by the purpose for which the data is required. The second GRDDC paper (Olusanya et al.) addressed a critical gap in the literature on the global and regional estimates of children with cerebral palsy and developmental intellectual disability based on the first-ever WHO-GBD Rehabilitation Need Estimator database. The third GRDDC paper (Olusanya et al.) summarized eligible systematic reviews and meta-analyses of the prevalence of eight prominent developmental disabilities published since the launch of the SDG in 2015. This systematic umbrella review underscored the limitations of traditional systematic reviews and meta-analyses for estimating the global prevalence of developmental disabilities as most of the primary studies were conducted in high-income countries.

Hadders-Algra reviewed the scientific justification for promoting school readiness within the construct of human brain development that emphasizes the role of early detection and intervention for optimal growth and development in LMICs. Nair et al. examined the concept, key dimensions, and evaluation of school readiness for children with disabilities based on an extensive review of the literature and highlighted the critical role of partnership among childcare givers within the health and education sectors in addressing the major challenges in promoting school readiness in LMICs. The paper clarified that school readiness requires targeted interventions for child readiness, school readiness and family and community readiness to facilitate inclusive education. Smythe et al. highlighted the critical role of culturally sensitive parenting interventions and related priorities to support school readiness for children with developmental disabilities in LMICs. Akhbari Ziegler et al. summarized evidence from two pilot studies in Brazil on implementing COPCA (COPing and CAring for infants with special needs), a novel, family-centered early intervention program for infants at high biological risk of neurodevelopmental disability. It can be delivered remotely through tele-coaching, thus overcoming the challenge of access to a physical facility commonly faced by families in LMICs.

Nanyunja et al. demonstrated the feasibility of an affordable, community-based, group, participatory, peer-led program of early care and support for young children (0–3 years) with developmental disabilities and their caregivers through a randomized control trial, as part of a school readiness initiative in Uganda. Breinbauer et al. identified surmountable challenges in serving the needs of children with disabilities under a national early childhood development initiative in Chile, and reported how targeted financial incentives to the education sector has facilitated access to inclusive education for children under 5 years with developmental disabilities. Samia et al. identified obstacles and structural challenges in promoting inclusive education for children and adolescents living with disabilities in Africa.

The articles in this collection complement other publications by GRDDC addressing the need for a disability-focused early childhood development program for LMICs (7–9), visionary global leadership and accountability for early childhood development initiatives by UN agencies (10, 11), and a robust funding and investment mechanism to support school readiness for inclusive education for children under 5 years with developmental disabilities in LMICs (3, 12). There is an on-going scoping review of the available guidelines by GRDDC to help care givers plan and deliver intervention services from birth against the backdrop of the different models, approaches and scope for early childhood development (13, 14).

In conclusion, the available evidence would suggest that the interest of children with developmental disabilities and their families will be better served by an independent global early childhood development initiative that seeks to optimize school readiness for inclusive education for children under 5 years in line with the commitment under the SDGs. This would require dedicated investment in family-centered early detection and intervention services and for supporting the transition of children with disabilities from home into pre-schools to enable stronger tripartite approach to “ready children”, “ready families and communities”, and “ready schools”. As we mark the mid-point of the SDGs this year, all stakeholders in the disability community must unite and leverage the commitment under the SDGs to make the vision of a purposeful early childhood development by 2030 a reality.
